# Self-Reported, Work-Related Injuries and Illnesses Among Restaurant Workers in Shiraz City, South of Iran

**DOI:** 10.5334/aogh.2440

**Published:** 2019-05-10

**Authors:** Mehdi Jahangiri, Fahimeh Eskandari, Narges Karimi, Soheil Hasanipour, Mahnaz Shakerian, Asma Zare

**Affiliations:** 1 Research Center for Health Sciences, Institute of Health, Department of Occupational Health Engineering, School of Health, Shiraz University of Medical Sciences, Shiraz, IR; 2 Research Committee, Department of Occupational Health Engineering, School of Health, Shiraz University of Medical Science, Shiraz, IR; 3 Gastrointestinal and Liver Diseases Research Centre (GLDRC), Guilan University of Medical Sciences, Rasht, IR

## Abstract

**Background:**

Restaurant sector is one of the most rapidly developing sectors in the world and there is evidence that restaurant industry has high levels of work-related diseases and injuries. This study examined the prevalence of self-reported work-related injuries and illness (WRIIs) and their association with demographic variables among restaurant workers in Shiraz, the capital of Fars Province, Iran.

**Methods:**

In this cross-sectional study, 300 randomly selected restaurant workers completed a self-statement, research-made questionnaire regarding the prevalence of self-reported WRIIs, in Shiraz, Iran. Statistical analyses were performed using SPSS version 20.

**Results:**

A high prevalence of work-related injuries (84%) and musculoskeletal disorders (70%) was reported among restaurant workers. Cuts and lacerations, arising from accidents with knives, were the most common injuries seen, followed by burns, falls, slips and trips. Moreover, the prevalence of occupational accidents had a significant association with work experience (p = 0.012), cooking (p < 0.001), as well as preparation and washing (p = 0.011). Age (p < 0.001) and work experience (p < 0.001) had a significance association with the prevalence of musculoskeletal disorders.

**Conclusion:**

Preventive measures and polices, through providing occupational health and safety services including trainings, personal protective equipment and health examinations, should be taken as to restaurants and catering industry in Iran.

## Introduction

Restaurant sector is one of the most rapidly developing sectors in the world. Restaurants employ a large number of people [1], while their safety and health have been neglected in Iran [2]. Due to the wide variety of work activities in the preparation, cooking, and distribution of food, restaurant workers are in danger of a wide range of occupational hazards, including falls, slips, burns, high temperatures, long working hours, standing work postures for long hours, walking long distances, and carrying heavy burdens in awkward postures. Restaurant workers have also been associated with increased risks for grease burns, fall accidents because of slippery kitchen floors, respiratory symptoms due to environmental smoke and cooking fumes, exposure to combustion-*derived* air toxics, explosion, and psychological risks [3,4].

Studies show that restaurant workers are exposed to various work-related diseases including occupational cancers, in particular lung cancer [5,6,7], blood and gastrointestinal diseases [8], musculoskeletal disorders [9,10,11,12], in addition to work-related accidents such as grease burns, equipment-based wounds, electrical devices, knives and blades, falls, and slips [13,14,15,16,17]. Many studies have been conducted on work-related injuries and diseases among restaurant workers in different countries. Carayanni in Greece found that 44.3% of restaurant workers had experienced at least one occupational accident and 56.7% had no idea about the dangers they were exposed to at their workplace [4]. The restaurant industry ranks the third among the United States industries for risk of worker injuries with 6.1 percent of work-related injuries between 2000 and 2008 [18].

However, there have been few studies in Iran about work-related diseases and accidents in restaurants. For example, Malakoutikhah et al. studied musculoskeletal disorders in wait staff in a study [19]. Owing to the contribution of food industry to job creation as well as provision of “nutritious foods” to meet the “dietary needs” and food security in other industries, supporting this industry is the first step in the industrial development of each country. Therefore, paying attention to the problems and work-related diseases of restaurant workers can contribute to economic growth of the country.

Because of all the above-mentioned points and lack of studies on work-related injuries and illness (WRIIs) of workers in Iran, the aim of this study was to provide a general picture of health symptoms and work environment characteristics as well as the prevalence of WRIIs among restaurant workers in Shiraz, capital of Fars Province.

## Materials and Methods

### Design and Samples

**Figure 1:**
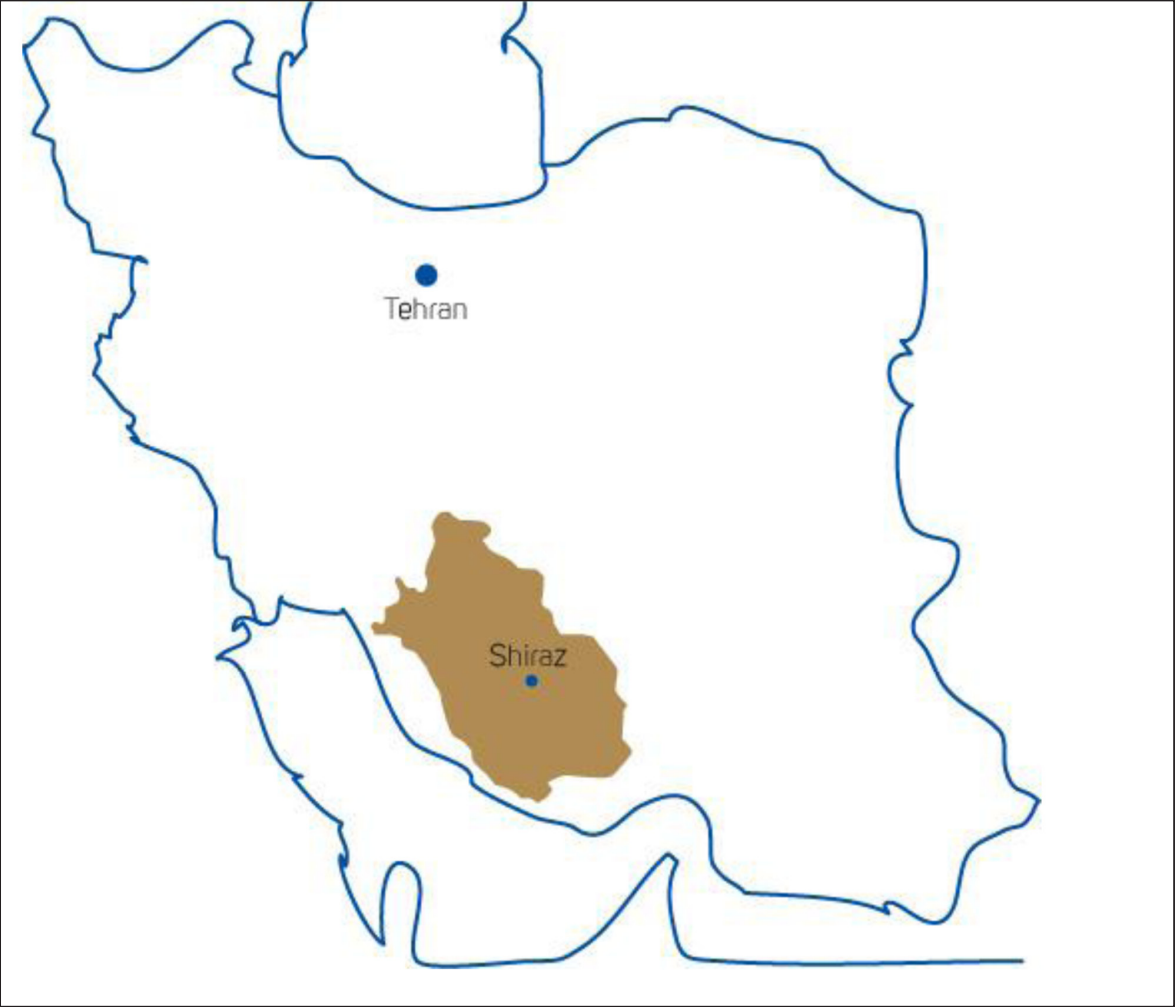
Map of the study areas.

This cross-sectional study was conducted among restaurants in Shiraz, the fifth-most-populous city of Iran and the capital of Fars Province, in 2017 (Figure 1). Sample size was calculated to be 367 by Epi Tools version 7 by taking 32% expected proportion of WRIIs [12], 95% confidence level, 5% margin of error and 10% attrition. For more confidence, the sample size was considered to be 375. One hundred restaurants were selected out of 1,303 in northern, southern, eastern, western, and central parts of Shiraz (12,387 workers) using a random number table. Study subjects were selected randomly among these 100 restaurants. The inclusion criteria were at least one year of work experience in different parts of the restaurant, except for administrative parts. Also, people who worked part-time in restaurants and had a second job were excluded.

Data was collected by researcher-made questionnaires containing questions about: A) demographic characterizes (including the size of restaurant, gender and number of workers, age, work history, job tenure, education level, marriage, type of employment); B) work-related injuries, the leading causes of injuries (fire and explosion, exposure to hot surfaces and steam, cuts and lacerations, electric shock, poisoning, falls, slips and trips, being struck by moving objects including hand tools, transmission vehicles), injured body part (Head/neck, Shoulder/arm/elbow, wrist, fingers, waist, hip, feet, knees, toes), and intensity of accident (low, medium, intense, very intense); and C) health symptoms of work-related illnesses in different body regions (eye, skin/hair/nail, nose/ear, cardiovascular, digestive, renal, respiratory, neurological, mental, end extremities, and musculoskeletal disorders, Table 1). Reported problems were limited to the past 12 months. The participants answered the questions by Yes/No.

**Table 1:** The content of diseases symptoms section of the questionnaire.

Work-related diseases/disorders	Symptoms

**Respiratory**	Cough, Phlegm, Shortness of breath, Wheezing
**Neurological**	Headache, Confusion, Vibration, Memory impairment, Seizure, Finger tingling
**Mental**	Excessive anger, Aggression, Anxiety, Low mood, Reduced motivation, Feeling of loneliness
**Circulatory**	Chest pain, Heartbeat, Shortness of breath at night, Shortness of breath in sleep mode, Bruise, Syncope history
**End extremities**	Tingling, White fingers, Numbness, Pain in fingers, Deformation
**Musculoskeletal**	Dry joints, Back pain, Knee pain, Shoulder pain, Elbow pain, Neck pain
**Eye**	Reduced visual acuity, Blurred vision, Eye fatigue, Diplopia, Burning eyes, Itchy eye, Fear of light, Tearing
**Skin/hair/nail**	Skin itching, Hair loss, Skin redness, Change in skin color, Chronic wound, Flaking, Change in the nail color, Dryness and cracking, Granules
**Nose/ear**	Hearing loss, Tinnitus, Dizziness, Ear pain, Ear discharge, hoarseness, Sore throat, Rhinorrhea, Olfactory disorder, Itching and burning nose, Nose bleeding, Dry mouth, Metallic taste in the mouth
**Digestive**	Anorexia, Nausea, Vomit, Stomachache, Stomach burning, Diarrhea, Constipation, Blood in the stool
**Renal**	Burning urine, Frequent urination, Bloody urine, Abnormal pain, Testicular mass

The formal validity of the questions was confirmed by 15 occupational health and safety experts. For this purpose, questionnaires were sent to experts by e-mail and they gave us their feedback regarding the necessary corrections. The questionnaires were used in pilot form, for a group of 30, and at intervals of two weeks. The percentage of agreement reached about 90% for respondents between the two measuring times, indicating high reliability of the questions. The data collection took four months. Confidentiality was maintained and informed consent was obtained. The workers were told that the collected data was just for the purpose of conducting a scientific study and they could discontinue participation in the study whenever they wished. During training of data collectors and supervisors, issues such as the data collection instrument, field methods, inclusion/exclusion criteria, and recordkeeping we emphasized. For those who were not literate, questionnaires were completed in interview form.

### Data Analysis

Statistical analyses were performed using SPSS version 20. Pearson chi-square tests were used to assess the univariate associations between the perceived variables and the reported WRIIs with highest of prevalence rate distribution, mean, standard deviation, and percentage were reported for each variable. The normality of each variable was then tested using Kolmogorov-Smirnov test with the error rate of ≥0.05. Chi-square and Pearson parametric tests were used to determine factors associated with WRII.

## Results

### Demographic Information

The response rate was 80 percent and 300 restaurant workers answered the questionnaire. The average amount of work experience and employment history of the restaurant workers were 10.28 ± 34.7 and 8.57 ± 10 years, respectively. The average number of workers in each restaurant was 25. About 30% and 70% of the subjects were working in fast food and traditional restaurants, respectively.

In this study, the association between demographic characteristics of restaurant workers and the incidence of work-related injuries and musculoskeletal disorders was investigated. Temporary worker doesn’t mean part-time worker, as mentioned in the excluding criteria; part-time workers have been excluded from the study. Temporary workers are defined according to their contract type. Workers with a permanent contract do not need to renew the contract periodically, but workers with a temporary contract may not extend their contract after one year and continue their work in the other restaurants. As shown in Table 3, type of contract could not affect the incidence of work-related injuries and musculoskeletal disorders among study population.

### The Prevalence of Work-related Injuries

**Figure 2:**
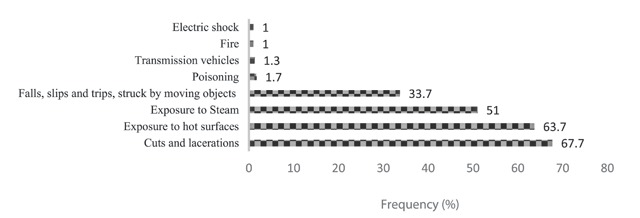
The prevalence of work-related injuries in the studied restaurant workers.

**Figure 3:**
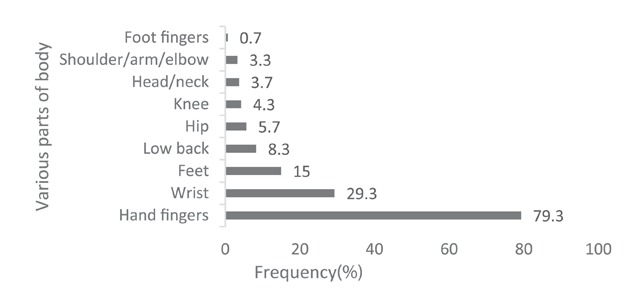
The prevalence of work-related injuries in various parts of the body in the studied restaurants workers.

As can be seen in Figure 2, cuts (67.7%), thermal burns (63.7%), scald burns (51%,) and falls, slips, and being injured by a moving object (33.7%) were the leading causes of work-related injuries in the restaurant industry. Figure 3 also displays the frequency of work-related injuries according to the part of the body injured in Shiraz restaurants. Most work-related injuries involved fingers (79.3%) and wrists (29.3%). The relationship between demographic characteristics and the prevalence of work-related injuries among the restaurant workers is presented in Tables 2 and 3. As shown, the prevalence of work-related injuries had a significant association with work experience (P = 0.012), cooking (P < 0.001) as well as kitchen workers (P = 0.011). But age, restaurant size, number of workers, level of education and type of employment had not a significant effect on the prevalence of work-related injuries (P > 0.05).

**Table 2:** Four demographic characteristics of restaurant workers and the association between the prevalence of work-related injuries and musculoskeletal disorders (n = 300).

Dependent variables			Work-related injuries		Musculoskeletal disorders	

Independent variables/number(percent)			Number (percent)	P*	Number (percent	P*

**Age (year)**	Under 20	11 (3.7%)	7 (2.3%)	0.085	1 (0.3%)	<0.001**
	21–30	116 (38.7%)	93 (31%)		69 (39%)	
	31–40	99 (33%)	88 (29.3%)		77 (25.7%)	
	41–50	46 (15.3%)	38 (32.7%)		39 (33%)	
	Over 50	28 (9.3%)	26 (8.7%)		25 (8.3%)	
**Work experience (year)**	1–5	108 (36%)	82 (27.3%)	0.012**	59 (19.7%)	<0.001**
	6–10	76 (25.3%)	63 (21%)		55 (18.3%)	
	11–15	61 (20.3%)	55 (18.3%)		50 (16.7%)	
	16–20	27 (9%)	24 (8%)		22 (7.3%)	
	More than 20	28 (9.3%)	28 (9.3%)		25 (8.3%)	
**Restaurant size (number of meals per day)**	Small (<150 meals)	29 (9.7%)	25 (8.3%)	0.08	21 (7%)	0.909
	Medium (150–500 meals)	117 (39%)	102 (34%)		81 (27%)	
	Large (500–1500 meals)	36 (12%)	25 (8.3%)		27 (9%)	
	Very large (>1500 meals)	118 (39.3%)	100 (33.3%)		82 (27.3%)	
**Number of workers**	1–5	71 (23.7%)	61 (21.3%)	0.14	44 (14.7%)	0.281
	6–10	80 (26.7%)	72 (24%)		59 (19.7%)	
	11–20	23 (7.7%)	19 (6.3%)		20 (6.7%)	
	21–30	25 (8.3%)	24 (8%)		18 (6%)	
	31–50	43 (14.3%)	31 (10.3%)		27 (9%)	
	51–70	27 (9%)	18 (6%)		20 (6.7%)	
	More than 70	31 (10.3%)	27 (9%)		23 (7.7%)	

* Pearson Chi – Square. ** P < 0.05 and had a significance difference.

**Table 3:** Three demographic characteristics of restaurant workers and the association between the prevalence of work-related injuries and musculoskeletal disorders (n = 300).

Dependent variables			Work-related injuries		Musculoskeletal disorders	

Independent variables/number(percent)			Number (percent)	P*	Number (percent)	P*

**Job title (job activities)**	Chef (cooking)	161 (53.7%)	154 (51.3%)	<0.001**	121 (40.3%)	0.057
	Kitchen workers (preparation, washing and cleaning)	98 (32.7%)	90 (30%)	0.011**	74 (24.7%)	0.181
	Delivery, warehousing	19 (6.3%)	6 (2%)	0.091	8 (2.6%)	0.99
	Waiter	22 (7.3%)	16 (5.3%)	0.347	19 (6.3%)	0.62
**Level of education**	Uneducated	10 (3.3%)	8 (2.7%)	0.821	7 (2.3%)	0.38
	Primary	43 (14.3%)	36 (12%)		32 (10.7%)	
	Diploma	214 (71.3%)	172 (60.7%)		153 (51%)	
	Academic	33 (11%)	26 (8.7%)		19 (6.3%)	
**Type of employment (contract type)**	Temporary	221 (73.7%)	185 (61.7%)	0.969	121 (43.7%)	0.542
	Permanent	79 (26.3%)	67 (22.4%)		58 (19.3%)	

* Pearson Chi – Square. ** P < 0.05 and had a significance difference.

### Prevalence of Work-related Diseases

**Figure 4:**
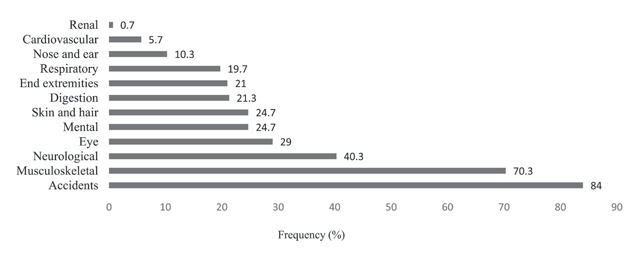
The prevalence of work-related injuries and illnesses in the studied restaurant workers.

In this study the prevalence of many diseases was investigated including renal, cardiovascular, nose and ear, respiratory, end extremities, digestion, skin and hair, mental, eye, neurological, and musculoskeletal diseases. As can be seen in Figure 4, renal diseases had the lowest prevalence rate (0.7%) and musculoskeletal disorders had the highest prevalence rate in the studied restaurant workers (70.3%). Because musculoskeletal disorders were the most prevalent among all diseases, these disorders are discussed in more detail, and due to space constraints, detailed discussion of all disorders was not possible.

**Figure 5:**
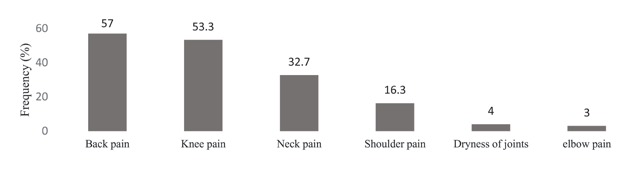
The prevalence of musculoskeletal disorders in the studied restaurant workers.

In Figure 5, the prevalence of musculoskeletal diseases in different parts was shown. Low back pain (57%), knee pain (53.3%), and neck pain (32.7%) were the most prevalent musculoskeletal complaints reported by the subjects, respectively (Figure 5).

## Discussion

The aim of this study was to investigate the prevalence of work-related injuries and diseases among restaurant workers in Shiraz city. The prevalence of work-related injuries among the workers was 84%. Cuts (67.7%), thermal burns (63.7%), scald burns (51%), and falls, slips, being injured by a moving object (33.7%) were the leading causes of work-related injuries among the restaurant workers. The highest and lowest prevalence rate of work-related diseases was related to musculoskeletal disorders (70.3%) and kidney and urinary tract diseases (0.7%), respectively.

Among work activities, distribution of injured persons was significantly higher in cooking processes (51.3%) followed by preparation and washing (30%) compared to other activities. This finding is consistent with the results of study of Jeong et al. (2015) conducted in South Korean restaurants where cooking (55.4%) and food preparation (38.4%) was the most common cause of occupational accidents [1]. This may be along with such risk factors as exposure to hot processes (hot grease and steam), handling knives and sharp blades, walking on slippery floors, and contact with hot dishes during washing and preparation activities.

As demonstrated in Figure 2, cuts, thermal burns, scald burns, and falls/slips were the most prevalent work-related injuries in the studied restaurant workers, respectively. The results of a study by Tsai et al. (2009), in which cuts were the most common incidents occurring to Chinese restaurant workers in the US, was also in line with our study results. Apart from the type of restaurant and type of work, the most frequent cause of these cuts was using knife and broken glass objects [20]. In this study, the use of knives and meat grinders was the most cause of cuts and lacerations, according the workers’ statements. However, only 19.3% of them used to wear protective gloves. Burns were the second leading cause of work-related injuries caused by working in warm environments, handling hot dishes, and exposure to hot grease and hot water, which is consistent with the study results of Tsai et al. [20] Uneven walking surface and inadvertent contact with objects were the most causes of slipping accidents, according to the participants’ statements.

In this study, the prevalence of work-related injuries for people with less than five years of work experience was significantly higher. This finding is consistent with those of other studies conducted in Japanese, South Korean, and Chinese restaurants [1,21]. Quick career change and short-term employment in restaurants make young workers unable to identify and control the workplace. On the other hand, in this industry, workers are rarely provided with occupational safety and health guidelines. As shown in Table 3, only 26.3% of the studied restaurant workers have been hired officially as permanent workers and 73.7% work in restaurants as casual workers with little work experience. Moreover, 40% of the subjects stated that they had not been trained regarding the principles of occupational safety and health.

In this study, low back pain (57%) was the most prevalent musculoskeletal complaint reported by the subjects. This is consistent with some of the studies conducted in other countries including those by Tomita et al. [22], Nagasu et al. [23] in Japan, and Chyuan et al. [24] in China. However, in the studies of Ihlebaek et al. [25] in Norway, Haukka et al. [26] in Finland, and Shiue et al. [27] in Chinese restaurants, shoulder pain and neck pain have been reported as the most common musculoskeletal disorders among restaurant workers, respectively. In general, the high prevalence of musculoskeletal disorders among restaurant workers has been attributed to the nature of work activities, inappropriate posture at work, long standing hours and carrying (manual materials handling) [12,22,26]. In this study, 93.3% of the subjects complained about prolonged standing and 98% stated that they, sometimes or always, had an inappropriate posture at work. Differences in the prevalence of musculoskeletal disorders in different countries can be attributed to different work processes and duties in various restaurants and countries as well as their effects on the body posture and stress experienced by the workers. For example, in a study conducted in restaurants with Western and Chinese foods, the highest prevalence rate of musculoskeletal disorders was found for low back (63.3%) and shoulders (62.3%), respectively [12].

In our study, the prevalence of musculoskeletal disorders was significantly correlated with the age and work experience of restaurants workers (Table 2). Contradictory results have been reported in studies investigating the effect of age and work experience on the prevalence of musculoskeletal disorders among restaurant workers. Some studies reported age and work experience as the risk factors for musculoskeletal disorders [28,29], while in other studies, musculoskeletal disorders were not significantly associated with age and work experience [23,30,31]. For example, according to Tomita et al. (2010), the prevalence of musculoskeletal disorders increased with increasing age among seafood processing factory workers [32]; however, in the study conducted on restaurant cooks in 2013, the prevalence of musculoskeletal disorders was not significantly associated with age and work experience [22]. The author attributed this to healthy worker effect. Miranda et al. have reported that the prevalence of musculoskeletal disorders increased after the age of 40 because of loss of muscular strength and the long-term effect of inappropriate postures at work [29].

According to the results of the present study, a significant prevalence of numbness and tingling in the extremities, especially in fingers, was reported in restaurant workers. This may be due to applying more force to prevent slipping knife while cutting, as well as repetitive motion and sustained static hand posture during cutting process [20]. In this study, 94.3% of the studied restaurant workers reported to have always or sometimes improper grip and discomfort in their fingers.

In this study, 44% of the restaurant workers reported suffering from headache, consistent with the studies conducted in Hong Kong and Portugal restaurant workers [12,33].

Factors such as food allergens, marine food toxins, disinfectants, and environmental factors, including heat stress and smoke, have been reported as the most important causes of headache among restaurant workers [34]. In this study, 66% and 94.4% of the subjects had sometimes or always been in contact with disinfectants and excessive heat, respectively. Moreover, 95% of the subjects had sometimes or always been exposed to smoke owing to various cooking processes such as barbecue, which increases the risk of carbon monoxide poisoning-induced cardio-myopathy from charcoal and leads to chronic headaches among restaurant workers [35].

In this study, 29% of the restaurant workers suffered from symptoms of eye disorders. Irritated (itching and burning) eyes, blurry vision, and eye fatigue were common symptoms reported in this study. The smoke of baking processes, high temperature and humidity, the use of spices, dust, the use of chemicals and detergents, and inappropriate lighting in some parts of the kitchen were the factors causing eye diseases reported by participants in the present study. However, only one out of the 300 subjects wore protective eyewear when using detergents. In a study conducted by Oliver et al. in UK catering staff, about 40 percent of the cooks reported eye discomfort, which is higher than this study. This difference is probably because of the use of incorrect type of lamps to electric fly killers and other devices that incorporate UV lamps which are not extensively used in the studied restaurants [36].

Studies have shown that working in restaurants has increased the risk of skin and hair disorders due to prolonged exposure to irritants (such as water and high temperatures) and allergenic factors (such as citrus fruits, spices, protein substances, and chemicals) [20,37]. In this study, the prevalence rate of work-related skin diseases in restaurants was 24.7%. Hair loss and *itchy, dry, and cracking* skin were reported as the most common symptoms. In a study by Gleeson et al., the prevalence rate of skin disorders in Irish restaurants was 15% [37], and according to Oliver et al., the prevalence rate of skin diseases in UK catering staff was reported to be 24% [36]. In our study, the prevalence of skin diseases was significantly associated with washing and cleaning works. High contact with detergents and disinfectants was the main cause of dermatitis in these workers. Skin cuts and scratches also increase the prevalence of fungal infections which are considered as biologic hazards because of too much humidity in restaurant kitchens. Contact with chemicals can be avoided by wearing protective gloves. However, only 19.3% of the studied restaurant workers used to wear gloves during their work.

In the present study, 24.7% of the restaurant workers reported psychological problems such as aggression and anxiety. High emotional demands in work with customers; shift work, night work, high work pace, long working hours, low control, low job security, and problems with coordination of work and family cause many mental health problems among restaurant workers [3,38,39]. Petree et al. (2012) showed that prevalence of psychological problems, including aggression and anxiety, was more frequent among restaurant workers who work long hours [38]. In this study, 69% of the studied restaurant workers worked long hours (more than 8 hours) and 52% were shift workers.

The prevalence rate of gastrointestinal diseases was 21.3% in the studied restaurant workers. Anorexia and heartburn were the most common symptoms of gastrointestinal diseases. Restaurant workers often eat their meals in the restaurant when there are few clients, not at the normal meal time. Moreover, along with the continuous access to food, they are constantly eating, and irregular eating habit is one of the main causes of digestive problems, especially anorexia [20].

In this study, the prevalence rate of respiratory problems was 19.7% among restaurant workers, which included dyspnea, wheezing, sputum, and cough. This finding is consistent with the results of other studies, including Juntarawijit et al. [2] and Adewole et al. [40], on restaurant workers in Thailand and Nigeria, respectively. The most important cause of respiratory problems among restaurant workers can be cooking fumes and the presence of organic compounds such as formaldehyde, acrolein, and Total Organic Compounds (TVOCs) as well as suspended *particulates* in the air inside restaurants [41].

There were some limitations in this study that should be taken into consideration when interpreting the results. The cross-sectional design of the study and the subjective nature or self-reporting of the collected data may not allow actual causative conclusions to be made. Furthermore, since the current research was conducted using a questionnaire for the last 12 months, recall error may have affected the results obtained. In addition, detailed information on the extent of injuries or damage of accident was not available in the source documents to ensure the answers.

## Conclusion

The results of this study indicated a high prevalence of work-related injuries (84%) and musculoskeletal disorders (70%) among restaurant workers. Therefore, preventive measures and polices, through providing occupational health and safety services including trainings, personal protective equipment, and health examinations, should be directed to restaurants and catering industry in Iran. Further research is necessary to more accurately determine the incidence, nature and leading causes of work-related injury and diseases in the catering industry in Iran. This would help the authorities in planning and implementing an effective health and safety management system.
